# Creation of a Systems-Level Checklist to Address Stress and Violence in Fire-Based Emergency Medical Services Responders

**Published:** 2019

**Authors:** Jennifer A. Taylor, Regan M. Murray, Andrea L. Davis, Lauren J. Shepler, Cecelia K. Harrison, Neva A. Novinger, Joseph A. Allen

**Affiliations:** 1 Drexel University Dornsife School of Public Health, Department of Environmental and Occupational Health, 3215 Market Street, Nesbitt Hall, Room 655, Philadelphia, PA 19104, USA; 2 Rocky Mountain Center for Occupational & Environmental Health, University of Utah Health, Salt Lake City, UT, USA

**Keywords:** Systems-level checklist, Workplace violence, ThinkLets, first responders, Emergency medical services, SAVER

## Abstract

Between 57 and 93% of Emergency Medical Services (EMS) responders reported having experienced verbal or physical violence at least once in their career. Therefore, the primary goal of this study was to develop a systems-level checklist for violence against fire-based EMS responders using findings from a systematic literature review and outcomes from a national stakeholder meeting. First, a literature review of violence against EMS responders resulted in an extensive list of 162 academic and industrial publications. Second, from these sources, 318 potential candidate items were developed. Third, Q-methodology was employed to categorize, refine, and de-duplicate the items. Fourth, ThinkLet systems facilitated consensus-building, collaboration, and evaluation of the checklist with diverse subject matter experts representing 27 different EMS organizations, government, academia, labor unions, and fire departments during a two-day consensus conference. The final SAVER checklist contains 174 items organized by six phases of EMS response: pre-event, traveling to the scene, scene arrival, patient care, assessing readiness to return to service, and post-event. So called pause points for the individual EMS responder were incorporated at the end of each of phase. Overall, 47.5% of votes across all phases rated items as most feasible, 33.7% as less feasible, and 11.6% as extremely difficult. The SAVER systems-level Checklist is an innovative application of traditional checklists, designed to shift the onus of safety and health from that of the individual first responder to the organization by focusing on actions that leadership can institute through training, policy, and environmental modifications.

## Introduction

***March 16***^***th***^**, *2017, New York, NY:*** *Yadira Arroyo, a 14-year veteran of FDNY was killed by a career criminal who commandeered her ambulance, ran her over, and dragged her beneath its wheels. Encountering violence in the field is not a rare occurrence for EMTs. In New York City, more than 100 members a year are assaulted on the job according to Robert Ungar, a spokesman for the Uniformed EMTs, Paramedics & Fire Inspectors F.D.N.Y. union*

(Mueller 2017).

***May 1***^***st***^**, *2017, Dallas, TX:*** *Paramedics responded to a call that purported a suicidal patient. Upon arriving on scene, EMS responders discovered a gunshot victim laying in the street and began to administer care. While providing life saving measures to the gunshot victim, the paramedic providing treatment was critically wounded by the armed gunman. The injured medic was in critical but stable condition after undergoing lifesaving surgery. The medic will require multiple surgeries and extensive treatment before a full recovery*

(McLaughlin 2017).

There were 29 million calls for Emergency Medical Services (EMS) in 2015, a 23% increase from 2014 (National EMS Information System 2016). This increase represents a continually growing trend in the United States. Fire departments provide about 40% of the nation’s EMS services (National Association of State EMS Officials 2011). In 2015, on average 64% of fire department 911 calls were for medical emergencies (National Fire Protection Association 2016), but can run as high as 90% (FIREHOUSE 2016).

Paramedics and EMTs believe they are under severe stress and are concerned about impacts on their mental health (Everline 2016; J. A. Taylor et al. 2016). However, such perceptions have not yet been systematically measured. In a previous study on their experiences with patient-initiated violence the following recollections were reported (J. A. Taylor et al. 2016):

“*I have been kicked, punched, bitten, spit on, verbally abused. You name it, I*’*ve had it all.*”

“*And [I] went to court, and this is where it’s disheartening, because that’s supposed to be felony assault*…*. And I’m wasting my time going to court two and three times…I knew there was no confidence in the system…I mean, you shouldn’t be able to do that to someone who’s trying to help you. Felony assault should stick.*”

The annual rate of non-fatal injuries among U.S. paramedics is five times higher than the national average for all workers (B. J. Maguire et al. 2005). The annual rate of occupational fatalities among paramedics is two times higher than the national average for all workers (Brian J. Maguire et al. 2002). In a retrospective cohort study of nationally registered U.S. EMTs, 8 % of fatalities were due to assaults (B. J. Maguire and Smith 2013). Compared to health care settings such as hospitals, workplace violence in EMS is inadequately described and requires further consideration (Koritsas et al. 2009; Koritsas et al. 2007; Terry Kowalenko et al. 2013; T. Kowalenko et al. 2005).

The National Institute for Occupational Safety and Health (NIOSH) lists 10 factors that may increase a worker’s risk for workplace assault, such as contact with the public, having a mobile workplace, working with unstable or volatile persons, working alone or in small numbers, etc. Eight factors apply to the EMS work environment (Jacob 2004). In an analysis of near-miss and injury events reported during EMS runs to the National Fire Fighter Near-Miss Reporting System, the most commonly identified mechanism of injury was assaults. EMS responders were threatened or assaulted by patients, family members, and bystanders. Patient factors included: drug/alcohol intoxication, mental illness, and underlying health conditions (e.g. seizure, hypoglycemia)(J. A. Taylor et al. 2015). In a study on fire service injury, paramedics experienced a 14-fold higher odds for violence injuries than firefighters (J. A. Taylor et al. 2016).

In a study of safety climate in fire departments across the United States, firefighters reported that the 911 system is strained due to the high volume of low acuity medical calls that occupy much of their workload, which divert resources from true emergencies and lead to unwarranted occupational hazards like speeding to respond to non-serious calls. As a result, firefighters reported high occupational stress, low morale, and a desensitization to community needs. Firefighters called for improvements to the 911 system including better triage, more targeted use of EMS resources, continuing education to align with job demands, and a strengthened social safety net to address the persistent needs of poor and elderly populations (Cannuscio et al. 2016). A critical barrier to progress in fire-based EMS is understanding the organizational, mental health, and safety burden that providers currently carry as they respond to an increasing community demand for services. There are concerns that this workforce is experiencing poor safety and organizational outcomes such as injury, depression, anxiety, PTSD, burnout, and decreasing job satisfaction. EMS responders have also reported higher rates of suicidal ideation and suicide attempts (Stanley et al. 2015; Stanley et al. 2016). These outcomes are very costly to employers and threaten the stability of the healthcare safety net EMS provides (Bigham et al. 2014; Federiuk 1992; Nordberg 1992; Patterson et al. 2010).

A systematic literature review of violence against firefighters and EMS responders in peer-reviewed and industrial journals (1978–2016) found that between 57 and 93% of EMS responders reported having experienced an act of verbal and/or physical violence at least once in their career (Taylor and Murray 2017). Up to the early 1990s, the issue of violence towards EMS responders was only discussed within industrial journals, underscoring the fact that industry understood the risks of the job long before the first peer-reviewed research was published in 1993. While prevalence estimates fluctuate slightly, authors are discussing the same issues 40 years later. No evidence-based interventions have been developed to support EMS responders from violence on the job, however industry articles provide potential organizational improvements that could mitigate the risks of assault including: policies for police backup; better information from the dispatch system including flags to identify previously violent locations; improved cultural competency, de-escalation, and body language techniques; personal protective equipment; and improved reporting of assaults (Nethercott 1997).

Given these best practices, the utility of a checklist that organizations can use to support EMS responders is plausible. Checklists acknowledge that accidents are inevitable in complex systems and work to reduce complexity (Perrow 1984). They have been used successfully in a myriad of industries to reduce errors, inconsistencies, and unsafe practice. For example, the field of aviation made strides in safety through the use of pilot checklists. From 1980 to 1996, 25% of fatal approach and landing crashes were due to performing the wrong action or omitting critical actions (Ashford 1998). To reduce such errors, pilots have access to brief but easy to read checklists such as the Northwest Airlines MD - 80 checklist (National Aeronautics and Space Administration 1990). Thus, the percentage of accidents due to human error decreased from 90 to 55 (Shappell et al. 2006). Other well-regarded checklists include: the Toyota Production System - a systems approach to define processes and needed communication coupled together to reduce confusion and encourage a culture of learning (de Bucourt et al. 2011; Institute of Medicine 2012; Spear and Bowen 1999; Toyota 2015) and the “Doctor’s Checklist” created at Johns Hopkins Hospital to avoid infections when inserting central lines into ICU patients (Berenholtz et al. 2004).

In EMS, there are numerous checklists for each phase of the call, such as the Responding to Violence Checklist by EMS Health & Safety (Collopy et al. 2011) or the Aggression Continuum by Steven Wilder and Chris Sorensen (Vernon 2009). None of the common practices were being codified into any standardized training at fire departments, meaning that first responders would need to self-educate in order to equip themselves with these skills. This gap in training and policy, coupled with the concerning levels of stress and violence experienced in EMS, initiated the need for development of a systems-level checklist.

The activity described herein is part of a Federal Emergency Management Agency (FEMA)-funded study, “Stress and Violence to Fire-based EMS Responders (SAVER).” The first of four study aims was to develop a systems-level checklist for violence against fire-based EMS responders using: (1) findings from a systematic review of academic and industrial literature and (2) outcomes from a national stakeholder meeting during which consensus on checklist items was achieved.

### Creation of the SAVER Checklist

The World Health Organization’s Patient Safety Programme developed the Surgical Safety Checklist using steps adapted from aviation (Weiser et al. 2010). The SAVER checklist, tailored to fire-based EMS responders, followed a similar development process including the steps of content and format, timing, trial and feedback (Weiser et al. 2010). Formal testing and evaluation, and local modification will be evaluated in future activities with fire departments participating in the SAVER program (Weiser et al. 2010).

The *Checklist Manifesto* by Atul Gawande (Gawande 2010) was a conceptual resource for the development of the SAVER systems-level checklist. However, most checklists currently in use – whether in healthcare (Weiser et al. 2010), nuclear power operations (Jou et al. 2009), aviation (Federal Avitation Administration 1981), or other high-risk industries – are focused on the **individual**, rather than on the **system** in which they operate:

Rather than being the main instigators of an accident, operators tend to be the inheritors of system defects. … Their part is that of adding the final garnish to a lethal brew that has been long in the cooking

(Reason 1990).

In the absence of systems thinking, there has been a tradition of blaming workers for their injuries. Even in cases of assault by patients, the fire and rescue service has few answers other than punishing the patient (e.g., felony conviction) or putting the onus back on the responder (“be careful,” “practice scene safety,” “it’s part of the job”). Systems thinking instructs designers to assume adverse events will happen and anticipate opportunities for prevention as upstream as possible from the interactions that cause harm. Such upstream opportunities are largely focused on organizational policies, procedures, and practices – as opposed to individual actions (Reason 2000). Based on our continuing work with the fire and rescue service, we wanted to propose a solution to work-related assaults that removes the burden from the individual-level responder and places it back on the level responsible for safety and health – the department and labor union. It is these organizations that make policy and create opportunities for training. Individual firefighters and medics do not. In our study of checklists, it seemed possible that a checklist could have benefit at the organizational level.

This is not to say that individuals do not have a role in preventing adverse events such as workplace violence. EMS responders are notoriously overworked with little time to think about organizational interventions (Cannuscio et al. 2016; J. A. Taylor et al. 2016). In addition, the hierarchical work environment of the fire and rescue service can make it hard for individuals to raise their voices. Gawande’s guidance to “push the power of decision making out to the periphery and away from the center” by employing checklists, ensures that the individuals with the least amount of power within an organization have the authority to make autonomous decisions that support their mission and safety (Gawande 2010). This translates into **pause points** - a set of discreet criteria that must be met before proceeding to the next task. A common example is the use of a “time out” at the beginning of a surgery whereby anyone on the team may raise concerns for the patient and the procedure about to be performed, which are then addressed before continuing (Gawande 2010).

## Methods

### Development of Candidate Checklist Items

**Fig. 1:**
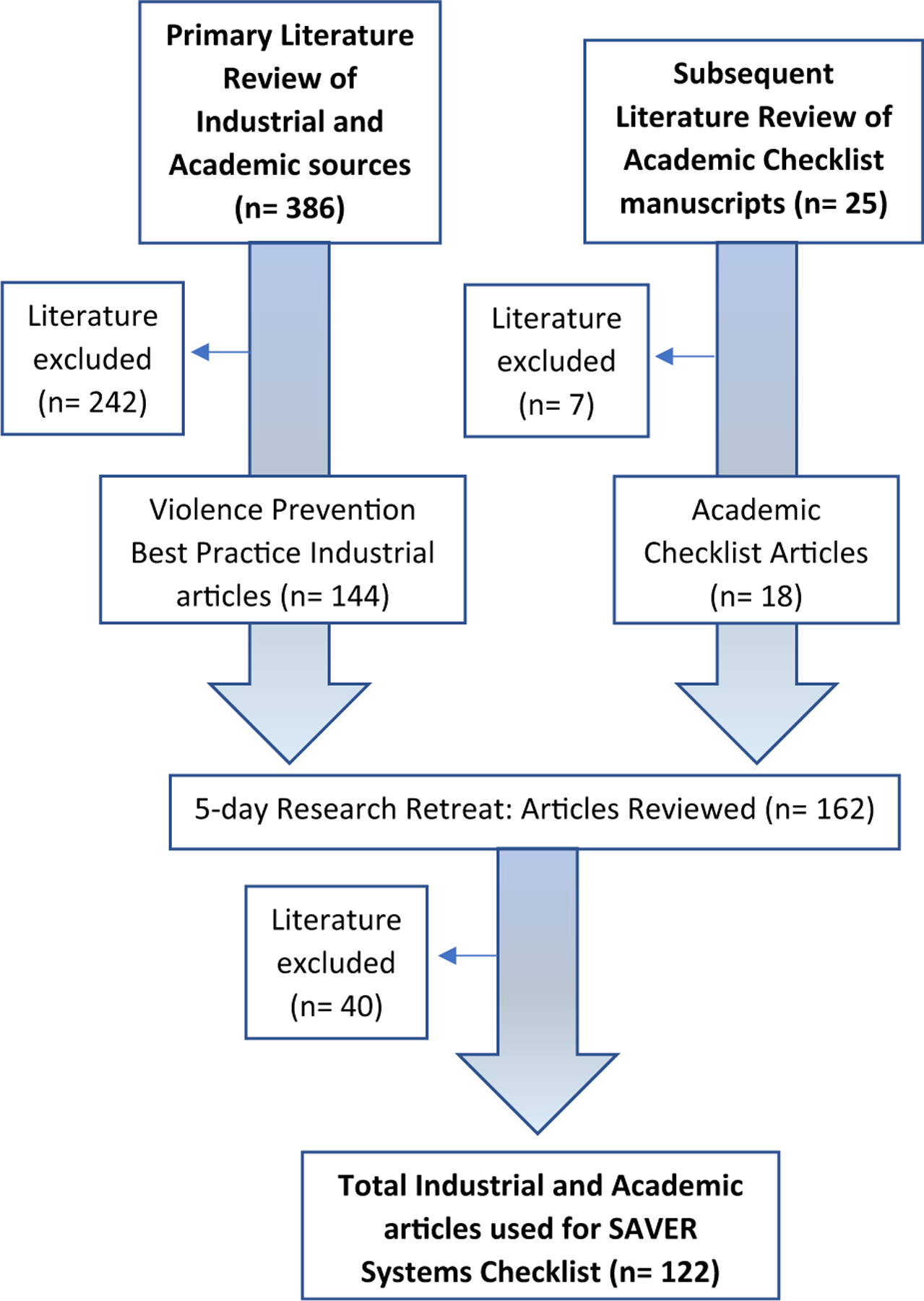
Flow Chart of Total Industrial and Academic Articles used for SAVER Systems Checklist

The primary literature review of violence against EMS responders was conducted previously for a commissioned report (Taylor and Murray 2017). That review had three phases. Phase 1 included articles reviewed based on title, abstract, and keywords; Phase 2 included article review, assessment, and documentation based on titles and abstracts; and Phase 3 included an in-depth analysis of all industrial and academic literature on the topic. Of 386 articles described in that report, a subset categorized as “preparedness and intervention” from industrial sources only (*n* = 144) were selected because they described best practices, policies, and training opportunities to prevent violence against EMS responders (Fig. 1). It was from these sources that the idea of a systems-level checklist emerged.

A subsequent search of the academic literature was conducted to describe the development, application, and evaluation of checklists. Search terms included: surgical checklist, aviation checklist, Atul Gawande, safety culture checklist, systems checklist, and safety domains. Of the 25 academic articles collected during this secondary literature search, 18 articles were selected for inclusion by CKH and RMM, because they discussed the development of systems checklists and organizational-level applications (Fig. 1).

From the above described literature reviews, a total of 162 manuscripts were compiled, read, and then analyzed chronologically during a five-day research retreat (CKH, LJS, ALD, RMM, and JAT). Further inclusion and exclusion criteria were discussed as a group to establish inter-rater reliability. Literature that did not suggest ideas for prevention that could be developed into a candidate checklist item were excluded after consultation with JAT and confirmation by RMM (*n* = 40). 122 articles were referenced for the development of candidate SAVER systems checklist items (Fig. 1).

Upon the conclusion of the research retreat, 318 candidate checklist items were developed. Each candidate checklist item was documented and assigned to the relevant phase of EMS response: “traveling to the event”, “scene arrival/prior to entry”, “patient care”, transport to the hospital”, “transfer to emergency department staff”, and “assessing readiness to return to service”. Next, Q-methodology, a systematic process for assessing subjective viewpoints often used in psychology and qualitative research, was employed. Q-methodology assists with the identification of similarities and patterns among sources, as well as their relationship to broader statements and categories (Shinebourne 2009). Each candidate checklist item was reviewed, the source verified, and then de-duplicated. Similar checklist items were grouped together based on content and co-author consensus. A total of 39 emergent themes were cataloged according to their goodness of fit within its assigned phase of EMS response or reassigned to the relevant phase. Then, all candidate checklist items were reevaluated for goodness of fit within each phase, and each theme.

Next, checklist items were focused at the level through which an organization (i.e., fire department and labor union) could support EMS responders. Items were drafted into one of four levels that emanated from the EMS literature: policy, training, technological or engineering modifications, and individual-level actions. From the initial 318 candidate items, a total of 159 checklist items and six pause points were developed. Themes were further refined based upon the development of the draft checklist items and team consensus (reduced from *n* = 39 to *n* = 25). Of the 159 checklist items, there were 78 policy items, 59 training items, 6 technological or engineering items, 16 individual-level items across the six phases of the checklist.

### Stakeholder Consensus Conference/Resultant Checklist

To provide the opportunity to review and revise the candidate checklist items, 41 diverse subject matter experts (SMEs) representing 27 different affiliations were invited to a two-day SAVER “Systems-level Checklist Consensus Conference (SC^3^)” in July 2018. The 41 SMEs included: 12 national fire service and EMS organizations, 6 individuals from federal, state, and local governments, 3 research and academic organizations, 14 fire department members, and 6 labor union representatives. In the United States, fire department labor unions are dues-paying membership organizations that advocate for firefighter and EMS responder employment benefits (i.e., work hours, wages, health and safety conditions) through collective bargaining processes. To ensure organizational and occupational representation from each of the four participating fire departments serving as SAVER study sites, one individual from each of the following fire department levels was invited: leadership, labor union, EMS field supervisor, and paramedic with 10–15 years of experience. Representatives from dispatch and law enforcement were also invited, though only 1 dispatch representative was able to attend. None of the attendees had previous knowledge of the items in any phase of the checklist, though all received a one-page document describing the SAVER program. SMEs were 78% male and 22% female.

**Fig. 2:**
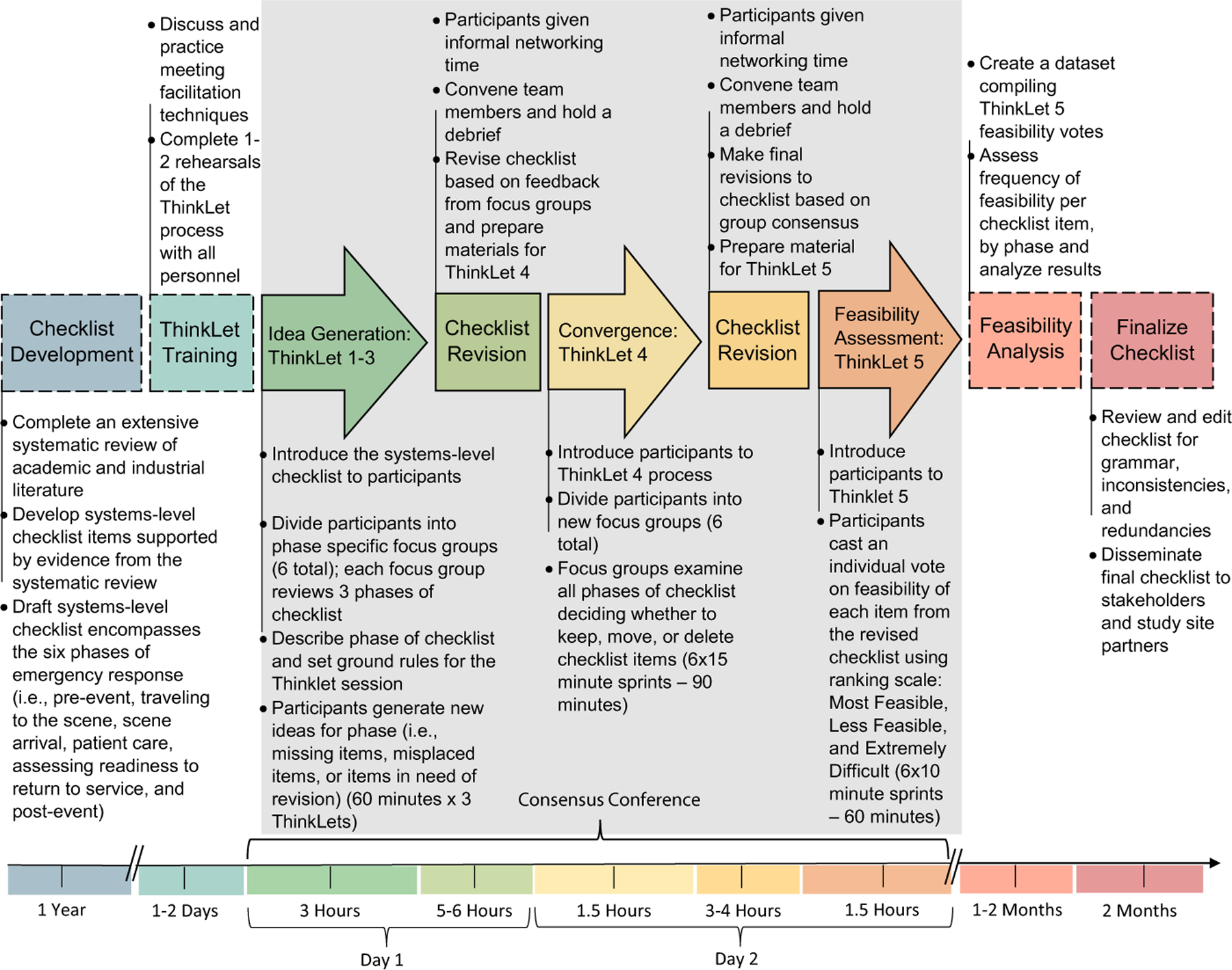
ThinkLet Process

We deployed a facilitated consensus-building collaboration method using a series of focus groups structured around three separate ThinkLet systems (De Vreede et al. 2006). The ThinkLet guides are available in the **appendix** and details on the ThinkLet process deployed are available in Fig. 2. As ThinkLet sessions were recorded, a human subjects protocol was submitted and approved by the Drexel University Institutional Review Board.

**Fig. 3:**
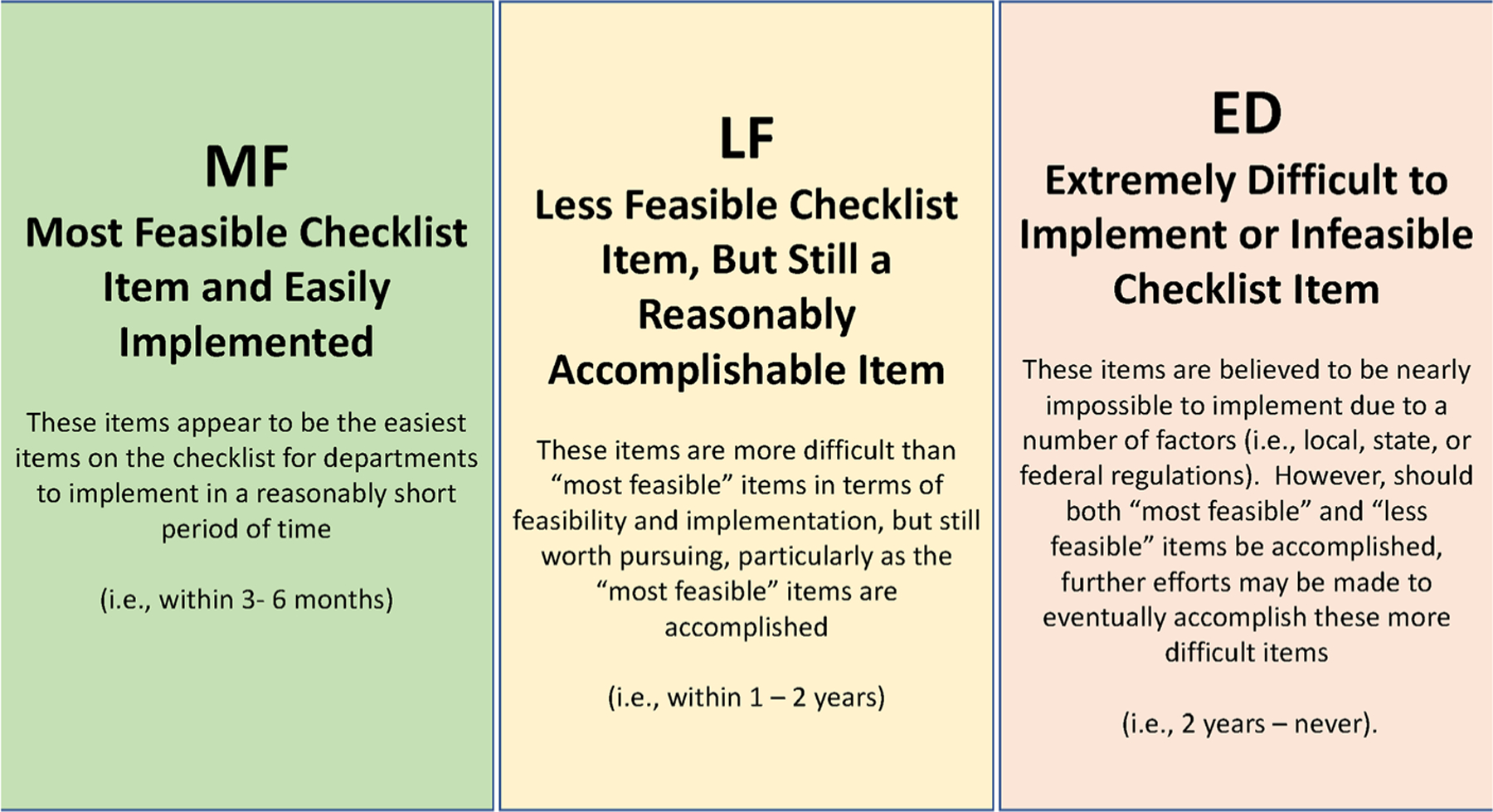
Rating Scale for Candidate Checklist Items

Through the ThinkLet process, the checklist was reviewed, revised, updated, and finally rated for feasibility. Following ThinkLets 1–3, the checklist grew from 159 to 242 candidate items. These items were reviewed by the co-authors who served as the facilitation team and preparations made to include all newly generated checklist items in ThinkLet 4 at the start of the next day. ThinkLet 4 processes led to the deletion of 10 candidate checklist items based on SME consensus. The resultant 232 candidate checklist items were voted on based on feasibility using three criteria: most feasible (MF), less feasible (LF), and extremely difficult (ED) (Figure 3). The feasibility assessment was completed individually to remove the potential for social desirability. Percent MF, percent LF, percent ED, and percent missing votes were calculated by total possible respondents and by each phase. For example, percent missing was calculated by the number of missing votes per checklist item, divided by the total possible responses (41) and divided by the total number of checklist items per that phase, multiplied by 100.

Further refinements to the resultant checklist were completed by JAT, RMM, and JAA. The resultant checklist was reviewed for grammar, inconsistencies, and redundancies and revised accordingly.

## Results

### Development of Candidate Checklist Items

The literature review process yielded 159 checklist items organized by phase of EMS response: “traveling to the scene,” “scene arrival,” “patient care,” and “assessing readiness to return to service.” Due to the paucity of literature and checklist items in the “transport to the hospital” and “transfer to emergency department staff” phases, these items were subsumed into the “patient care” phase. Two additional phases, “pre-event” and “post-event” were developed based on the team’s analysis of gaps in the literature and results from the co-authors’ previous EMS research.

The “pre-event” phase focuses on the organizational structure (e.g., a fire department and its labor union). It is intended to help these organizations work together to develop policy and create training curricula that address EMS stress and violence. This enables and supports two goals: safe workers and quality patient care. For example (Table 2):

1.3: Does your department train EMS responders for potential verbal and physical violence (examples include: prevention, patient abandonment, felonious assault laws, cultural competency, simulation, self-defense, law enforcement cross-training, fit-for-duty, etc.)?
1.5: Does your department express through policy that verbal and physical violence against members is not tolerated?

The “post-event” phase emerged from the realization that while there are many tasks that need to be completed after an EMS run (e.g., typing patient care reports, cleaning and restocking the ambulance, communicating availability to dispatch, bathroom, and food breaks), there was no point in the phases of response that gave EMS responders protected time to document and report violent encounters, or reach out for mental health support. If encounters are not documented, evidence to describe the extent of the stress and violence problem does not exist. Furthermore, opportunities for recovery and treatment of physical and psychological injuries cannot be addressed. For this reason, the post-event phase has a strong emphasis on mental health impact and recovery, as well as a feedback mechanism for what is not working in the field. For example (Table 2):

6.1.f: Does your department have a policy that protects an EMS responder’s time -either by going out of service or using overtime - so that they can easily report any acts of violence or exposure that they experienced on a call, before they return to service and go on their next call?
6.15: Does your department’s training curriculum recognize and train on stress as a chronic occupational exposure including the relationship between the EMS responder workload and its cumulative stress impact?

Following the consensus conference, these new phases of emergency response remained on the checklist, confirming need, acceptance, and relevance to EMS work by the SMEs.

### Pause points

Using the previously described systems framework, draft checklist items were developed at the following levels within each phase of response: policy, training, technological or engineering modifications, and individual-level actions. Few individual-level actions were identified. Six of these were designated as **pause points** (Gawande 2010) because they focused on potential individual actions that could be taken at critical decision points to protect the responder’s safety. Pause points provide responders with the authority and autonomy needed to engage in safe patient care, provide a protection of self, and engineer an opportunity to give feedback to the organization about what is not working in the field. This results in a flipping of the traditional paradigm from placing the onus for safety on the responder and moving it back onto the organization:

- Traveling to the scene: If you have knowledge that this is a previously known violent location, request and wait for law enforcement backup.
- Scene arrival: Before exiting the ambulance, are all of the resources you need in place to safely begin patient care?
- Patient care: Before transport, does your patient require restraint and have they been checked for weapons?
- Assessing readiness to return to service: Are you mentally and physically ready to return to service?
- Post-event: If you have experienced verbal or physical violence, have you utilized the appropriate method for reporting?
- Post-event: Have you sought and received the physical and long-term mental health resources you feel will enable you to return to work whole and ready?

### Outcomes from the SAVER Systems-Level Checklist Consensus Conference (SC^3^)

#### Feasibility Assessment

**Fig. 4:**
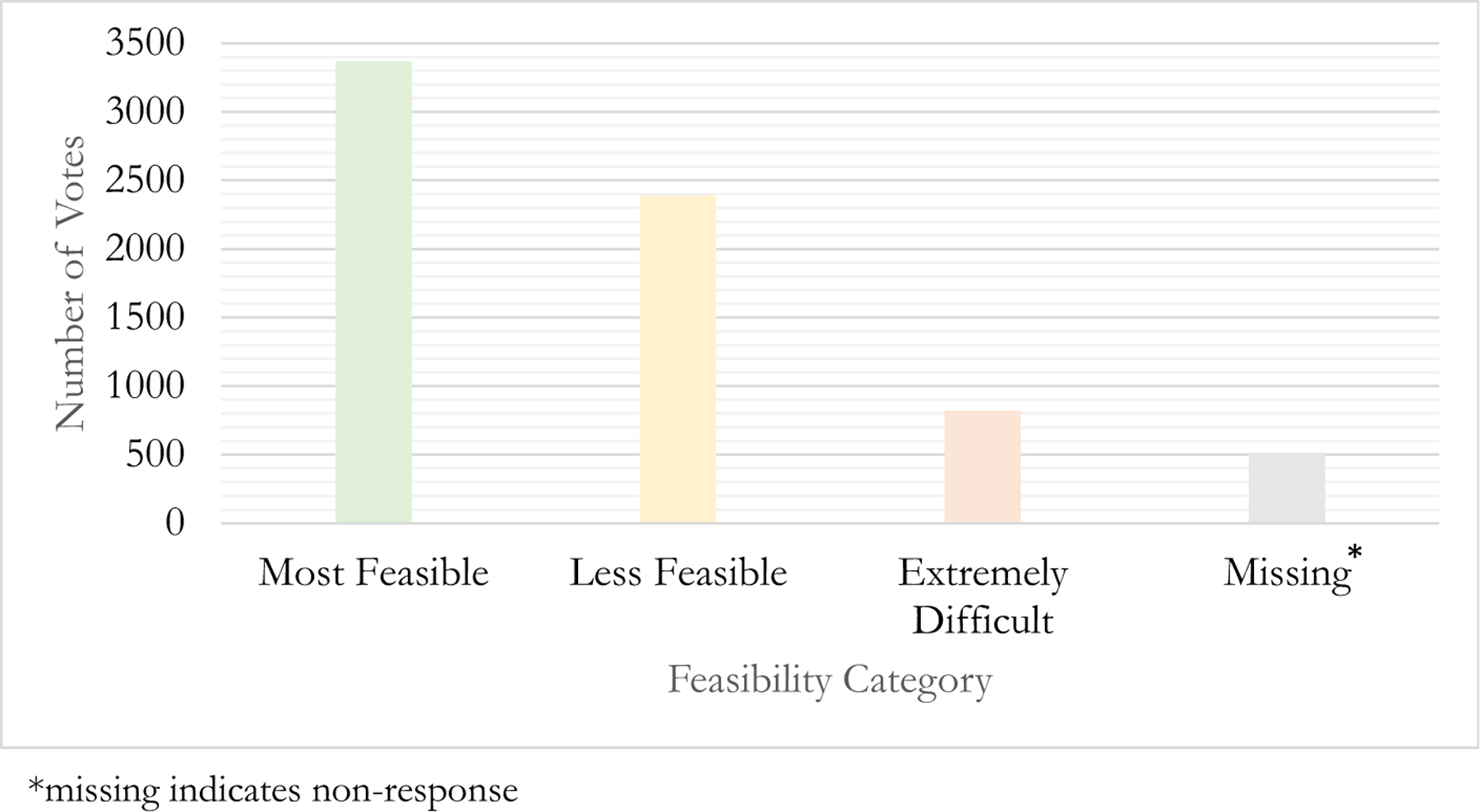
Checklist Feasibility Distribution by Number of Votes

Overall, 47.5% of votes across all phases rated items as most feasible, 33.7% as less feasible, and 11.6% as extremely difficult. (Fig. 4, Table 1). “Assessing readiness to return to service” was the only phase in which the majority of votes rated items as less feasible (39.8%). This phase also had the highest percentage of votes rating items as extremely difficult (19.8%). Missing votes across the phases ranged from 5.3–13.3%.

The final SAVER checklist (Table 2) contains 174 items organized by six phases of EMS response: “pre-event” (*n* = 41), “traveling to the scene” (*n* = 16), “scene arrival” (*n* = 14), “patient care” (*n* = 30), “assessing readiness to return to service” (n = 30), and “post-event” (*n* = 43). Pause points (*n* = 6) for the individual EMS responder were incorporated at the end of each of phase. Analysis of the final checklist shows that 70% of the items were from the original candidate items developed by the research team during the literature review process, and 30% of the items came from new items generated at the conference. Thus, the final checklist as reported here includes comprehensive scientific and industrial literature as well as integrated subject-matter expertise from the field.

## Discussion

High-reliability organizations, such as EMS, are expected to operate in complex environments that have a high risk for occupational illness and injury (Weick and Sutcliffe 2007). The risks that an EMS provider is exposed to on each run has the potential to be career or life-ending. The increasing number of calls in the United States adds to an already significant emotional demand inherent to health care professions. At the systems-level, organizations have a responsibility to develop policies and training that support and confer competencies necessary to effectively perform the job of an EMS responder. The SAVER systems-level Checklist addresses such opportunities by pointing organizations to the resources, tools, and knowledge they need to support the ever-changing mobile work environment faced by their EMS responders. The SAVER Checklist has two goals: (1) fire departments and labor unions should immediately use the systems-level checklist to assess and then plan for implementation of the items – starting with what is most feasible, and (2) these organizations should empower EMS responders to use the pause points (individual-level checklist) to protect their health and safety while on calls. The organizations should then act on feedback emanating from members in the field. To uphold the importance of EMS responder feedback, organizational leaders are to ask the EMS responders what the facilitators and barriers to effective implementation are for each checklist item.

The SAVER systems-level Checklist took what industry journals have been saying for 40 years - that violent events to first responders can be mitigated - and created a checklist for the *system*, instead of a checklist which would put more burden on already overstretched responders.

The SAVER checklist is innovative and different from other checklists because of its focus at the *organizational* - rather than the individual - level. If organizations are truly concerned about the health and safety of their members, they will demonstrate such through the development of meaningful policy and ongoing training. The individual-level pause points confer autonomy and authority to individual responders that allow them to make decisions that prioritize their safety. In so doing, the organization is creating a supportive environment that can further positively impact organizational outcomes such as burnout, job engagement, and job satisfaction, as well as decreasing the number of assaults and injuries experienced by EMS personnel.

The feasibility results from the consensus conference provided interesting findings, some of which surprised our research team. For example, 47% of the votes rated items as most feasible signifying that our diverse SMEs believe that these checklist items could be implemented in 3–6 months. That 33% of the remaining votes rated items as less feasible, but feasible within 1–2 years was also encouraging. These assessments can help fire departments prioritize the most feasible items and then move on as time and resources allow to those rated as less-feasible.

The “pre-event” through “patient care” phases were rated as most feasible. Pre-event is a new phase of the response process developed by our research team and underscores the importance of the *structure* of an organization, specifically, a fire department. What policies, procedures, practices, and training opportunities should be present in order to ensure the health and safety of EMS responders? What technology or engineering innovations are needed in order to keep them safe on the job? And what can the organization do to ensure their safety and health while they are on a call so that they can focus on quality patient care?

All pre-event items originally generated by the authors during the literature review phase were included in the final checklist (*n* = 22), along with an almost equal number of new items developed by the conference participants (*n* = 19). Fifty one percent of the votes described items in this phase as most feasible, which is inspiring since they are largely focused on policy development. We would have expected less enthusiasm more in line with that expressed in the “assessing readiness” and “post-event” categories. If fire departments and their labor unions can focus their efforts in the pre-event phase where they rate the action items to be most feasible, then there is a potential for mitigating even initial exposure to violence for EMS responders.

Development of the pre-event phase affirmed our use of a systems approach in that there are policies, procedures, and practices that departments can create before an emergency response even begins. Pre-event is supported by research on safety climate which stresses upstream intervention in the form of policy and training that will reward and support individual safety behaviors and therefore prevent undesirable outcomes like occupational injury (Beus and Payne 2010; Christian et al. 2009; Huang et al. 2016; Nahrgang et al. 2011; Zohar 2010). The more organizations focus on the pre-event phase, the more focused they are on prevention.

The last phase of the checklist, the post-event, was developed by our team as well. Traditionally departmental policies and procedures have emphasized what responders should do when on a call itself, but after the job is done there are many tasks that should be completed to return EMS responders back to the field whole and ready for the next call. For example, reporting injuries, assaults, or experiences of verbal violence to supervisors; the need for mental health support and rest and recovery from this highly emotionally demanding job; and a feedback mechanism for what is not working in the field. As the post-event phase is focused on protected time for reporting and pursuit of mental health resources, additional momentum and cultural shifts will need to continue in order for the fire service to fully embrace this phase of the checklist (see NLSI #12 and 13 (National Fallen Firefighters Foundation 2004)).

The phases “assessing readiness to return to service” and the “post-event” phases were rated as less feasible. Given that these phases had little to no evidence in the academic or industrial literature, it is not surprising that our SMEs rated these items as “extremely difficult” more so than other categories. These phases likely represent where the current system is breaking down or needs more development. This is simply part of continuing the fire service evolution from that of a reactionary culture (i.e., waiting until something bad happens to address safety concerns) toward an increasingly proactive culture. These items are largely more difficult to implement because they involve organizational changes, behavioral changes, increases in staffing, and additional resources that are already stretched thin in most fire and rescue departments. To this point, because the checklist’s length could present a realistic impediment, we suggest that fire departments start with what is most feasible and then plan for less feasible and extremely difficult items to be addressed over time. We are also investigating reducing the amount of items in each phase of the checklist by selecting the items that most support operationalization of the pause points as initiators for organizational change.

## Limitations

Although the development of the checklist was an essential first step to improving the safety and well-being of EMS responders, the development process was not without limitations. First, one goal of the consensus conference using the ThinkLet process was to reach data saturation on the potential checklist items. Further, our team selected three rounds of Item Generation ThinkLets due to time constraints within a two-day conference format and supported by data saturation science, specific to qualitative research methods and ThinkLets (Steinhauser et al. 2008; Tracy 2013). Though the number of new ideas toward the end were fewer and we feel we were close to saturation, additional ThinkLet sessions would have reached complete saturation. We are confident that what is presented is comprehensive and complete.

Second, the purpose of ThinkLets 1–3 was to generate ideas not present or missing from the draft checklist. As such, these newly generated ideas were generally not written into formal checklist items. While time between Day 1 and 2 allowed the authors to review and incorporate the newly generated checklist ideas, time constraints limited the authors’ ability to significantly process and refine all checklist ideas into formal, polished checklist items (a process candidate items enjoyed). Thus, as a check to verify that the new items were not rated as either more (e.g. because the SMEs like them) or less (e.g. because they were not as polished) feasible was performed. The overall feasibility results for the original items versus the new items from the conference did not differ significantly. That is, the pattern of most feasible, less feasible, and extremely difficult was essentially the same for both groups of items on the final checklist (i.e. 50%, 30%, 12%, with 8% missing; data not shown, but on request available from the first author). This helped us feel confident that biases in the ratings were neither present in the feasibility assessment, nor in the final checklist as currently constituted.

Third, due to unexpected resource limitations related to ThinkLet 5, we adjusted the process and implementation of the feasibility assessment. Specifically, instead of open voting visible to everyone at the conference, we initiated a closed ballot approach. Interestingly, this conferred a strength, in that it eliminated the potential for participant responses to be altered by social desirability when discussed in a group setting; however, because these new processes were not planned nor rehearsed, opportunities to field test and revise the formatting of the checklist were missed, and may have contributed to the resulting missing data from ThinkLet 5 (i.e. participants could skip rating items where desired). Other potential reasons for missing data include: time constraints and phrasing of some checklist items as an “if/then” statement; if the stem item was not voted with a high regard to feasibility, then the subsequent “if/then” root item was largely skipped.

Fourth, while the modified feasibility assessment in ThinkLet 5 was re-designed to be anonymous, in retrospect we believe it would have been beneficial to capture the participants’ identity during this ThinkLet session. Without these data, we are unable to stratify responses based on organization or rank within a fire department. It is possible that members of fire departments were more likely to rate feasibility with more certainty than others from outside the fire service. Thus, our ability to make conclusions from the feasibility assessment that would be impactful for checklist implementation processes are limited. To address this issue, we will conduct an additional feasibility assessment prior to checklist implementation at our study sites. Moreover, because the primary focus of the ThinkLet process was to establish consensus on the feasibility of the checklist, we did not assess participants’ valuation regarding the criticality or importance of each checklist item. Future work will include asking our study sites to review and rate the checklist items by the importance of the item for implementation and see how these ratings relate to feasibility. We will also measure if any checklist items are currently in use. Despite these limitations, we are confident our actualized methods of ThinkLet 5 resulted in an improved and streamlined process.

Finally, our exceptional panel of SMEs did not include some of the expert perspectives we had hoped to include, such as more 911 dispatchers and law enforcement. Despite this, we feel confident that key stakeholders perspectives were represented in the process of developing the checklist shown here. Through this publication, we invite those who have ideas and feedback on the checklist to contact the corresponding author.

## Future Use, Implementation and Validation

By utilizing the highly efficient ThinkLet process, key SMEs in the field of EMS were able to come to consensus within a two-day conference format to ensure the SAVER systems-level checklist was comprehensive for all phases of EMS response. With a focus on systems-level actions, the checklist will require leaders and representatives in both fire departments and labor unions to work collaboratively to maximize the acceptability and impact of the checklist upon implementation. The checklist should be used to promote dialogue and goal setting over time. As all checklist items have been developed within a systems framework, organizations must assess the facilitators and barriers to implementing each checklist item and incorporate this into their plan. This important dialogue will be crucial to the overall impact and support felt by the front-line providers.

The SAVER systems-level Checklist is an innovative application of traditional checklists, designed to shift the onus of safety and health from that of the individual first responder to the organization. The individual EMS responder does not use the systems level checklist. The only thing they need to do is use the pause points (an individual-level checklist) to provide feedback and stop ongoing processes from hurting them. Therefore, while the organization is responsible for 174 checklist items on the resultant SAVER checklist, the individual EMS responder is responsible only for 6. The SAVER checklist is predominately focused on actions that the leadership team (the fire department and labor union) can institute through training, policy, and environmental modifications. The pause points act as feedback loops for the individual EMS responder. This distributes the power throughout the hierarchy of the organization and gives the individual responder the autonomy and authority to pause mid-response at multiple time points, should they determine there are risks or threats to their personal safety. The training and policy instituted at the department-level will give first responders the tools to know how and when to call a ‘time out’ at these specific pause points to maintain their safety while responding. It is interesting that when the 174 final checklist items were redistributed among the domains of policy, training, environment, and individual, all of the original numbers stayed the same with the exception of policy. That category increased from the 78 draft items to 109 after the subject matter experts gave their input. The authors believe this is an expression of the importance of policy as an emergent need in the United States fire service.

While the checklist was developed with fire-based EMS organizations in mind, it holds relevancy to the private EMS sector, as well as small, rural, and volunteer departments. Representatives from these sectors were present at the consensus conference, thus we are confident that the checklist will have impact for various models of EMS. Upon completion of an additional feasibility assessment with the fire department study sites, the checklist will be tested with four large-metropolitan fire-based EMS departments. This implementation will include a battery of psychological tests to examine the degree to which the organizational-level intervention is impacting the well-being of employees. The implications for EMS responders are clear in that if the intervention works as designed, we would expect to see decreasing levels of burnout, decreasing assaults and injuries, and increasing job satisfaction and engagement with work. So, while the checklist is organizational in implementation, the result is impact on the individual worker.

This checklist was the result of a significant collaboration between the subject matter experts and the research team. In addition to understanding what is immediately achievable versus what remains long-term, it is important to acknowledge the emotions driving this work. The EMS side of fire has long been ignored. Its hazards from violence poorly described and tracked. Therefore, the importance of this work to our SMEs is best memorialized in their own words: “It’s about time someone cared enough to do something!”, “I feel this is the turning point to provide funding, resources, and a voice to people who need it.”, and “This subject has been long overdue. The environment is not getting any safer, the streets are getting more dangerous.”

## Supplementary Material

ESM1

ESM2

ESM3

## Figures and Tables

**Table 1:** Feasibility assessment of checklist items (n = votes)

Phase of EMS Response	Most Feasible % (n)	Less Feasible % (n)	Extremely Difficult % (n)	Missing* % (n)
Pre-Event	**51.1% (859)**	29.7% (499)	12.1% (203)	7.1% (120)
Traveling to the scene	**56.1% (368)**	21.6% (142)	9.0% (59)	13.3% (87)
Scene Arrival	**65.2% (374)**	22.6% (130)	4.2% (24)	8.0% (46)
Patient Care	**51.1% (629)**	34.7% (427)	6.6% (81)	7.6% (93)
Assessing Readiness to Return to Service	34.3% (408)	**39.8% (473)**	19.8% (235)	6.1% (73)
Post-Event	**41.5% (732)**	**40.8% (719)**	12.4% (218)	5.3% (94)
Total Votes	47.5% (3370)	33.7% (2390)	11.6% (820)	7.2% (513)

Bolded numbers highlight distribution of within category majority votes

* missing indicates non-response

**Table 2:** “SAVER Systems-level Checklist”

Checklist Number	Phase 1. Pre-Event	M.F.	L.F.	E.D.	Missing*
*Mission*					
1.1	Does your department have as part of its mission statement (i.e., core values, vision, organizational philosophy, etc.) that the safety and health of its members is paramount in order to provide quality community service?	37	1	1	2
1.1.a	Does the department have as a part of its mission statement the expressed recognition and commitment to emergency medical services?	39	1	1	0
1.2	Does your department implement policies, practices, and procedures that support EMS responder safety?	23	8	1	9
1.2.a	Does your department utilize clear definitions of violence, both physical and verbal? Does this include exposures related to violence (e.g., bites, bodily fluids)? Does your department provide training on all definitions?	24	15	1	1
1.2.b	Does your department have a policy for if and when EMS responders can use self-defense or other means for protecting themselves?	25	10	6	0
1.3	Does your department train EMS responders for potential verbal and physical violence (e.g., prevention, patient abandonment, felonious assault laws, cultural competency, simulation, self-defense, law enforcement cross-training, fit-for-duty, etc.)?	14	20	4	3
1.3.a	Does the training include hands-on self-defense instruction?	8	17	9	7
1.3.b	Does the training include the development of situational awareness?	22	11	1	7
1.3.c	Does the training include how to use department approved protective and defensive equipment?	11	13	10	7
1.4	Does your department encourage a level playing field (i.e., flattened hierarchy) among ranks when expressing safety concerns?	27	5	4	5
1.4.a	Does your department encourage a speak-up culture surrounding verbal and physical violence, without fear of harassment, embarrassment, or punishment?	20	10	3	8
1.4.b	Does your department cultivate a team-centric approach to patient care (i.e., EMS responders, firefighters, dispatchers, and leadership as equal participants of the team)?	27	9	4	1
1.4.c	Is there a standing EMS or labor management committee that regularly meets to discuss responder safety issues?	26	10	3	2
*Zero-Tolerance for Violence*					
1.5	Does your department express through policy that verbal and physical violence against members is not tolerated?	23	12	6	0
1.6	Does your department utilize a placard on the vehicle to educate the public that it is a crime to assault an EMS responder (if the law exists in your state)?	20	16	4	1
1.6.a	Does your department display placard in patient native languages (e.g., Spanish)?	14	11	6	10
*Department Practices*					
1.7	Does your department have a psychological evaluation as part of the hiring process?	11	18	12	0
1.8	Does your department select uniforms that clearly designate and separate EMS responders from other first responders (e.g., police)?	22	18	1	0
1.9	Does your department have a policy that dictates who may ride with the EMS responder?	35	4	1	1
1.9.a	Does your department have a policy concerning who may ride in the patient care compartment of the ambulance?	33	5	1	2
1.9.b	Does your department have a policy concerning who may ride in the cab of the ambulance?	33	5	1	2
1.10	Does your department have a policy or procedure that outlines when police should escort an ambulance with a violent or arrested patient?	28	9	3	1
1.11	Does your department have policies for securing patients for their safety and the safety of the responder?	37	3	0	1
1.12	Does leadership in your department ride with EMS responders to have a thorough understanding of their work environment?	27	12	1	1
1.13	Does your department provide ride-alongs and fire/EMS 101 for local politicians, media, researchers, clinicians, etc.?	19	15	6	1
1.14	Does your department have a policy to cross-train with other agencies regarding violence (e.g., Police, Dispatch, Social Work, Community Health, etc.)?	10	22	8	1
1.15	Does your department provide training for dispatchers on recognizing when to flag calls as specific types?	23	10	5	3
*Professional Behavior*					
1.16	Does your department have policies regarding professional behavior and communicating with patients, patient families, and bystanders as a de-escalation technique for EMS responders and dispatchers?	29	9	1	2
1.16.a	Does your department have trainings and simulations for EMS responders and dispatchers on professional behavior?	12	21	4	4
1.17	Does your department have de-escalation training for mediating violent acts?	16	21	3	1
*Communication*					
1.18	Does your department have a universal code (e.g., mayday) for distress or emergency for EMS responders?	28	10	1	2
1.18.a	Does your police department use the same universal code?	16	16	5	4
1.18.b	Does the municipality use the same universal code?	11	21	5	4
1.18.c	Does the state use the same universal code?	0	18	18	5
1.18.d	Does the entire national EMS services system use the same universal code?	0	10	27	4
1.19	Does your department have the ability to monitor and record violent events in the field (e.g., black box, body cameras, physiological monitoring, etc.)?	8	18	14	1
1.20	Does your department have a means of communicating between the cab and patient care compartment of the ambulance (e.g., window, headset, radio in windowless, video camera display, etc.)?	26	14	0	1
1.21	Does your city use billboards to display EMS personnel working to care for the public and remind the public to be kind when help arrives (e.g., care for those who care for you)?	3	22	15	1
1.22	Does your department have interagency protocols or agreements for communication and data sharing? (e.g., Law enforcement, mutual aid, etc.)	23	13	2	3
1.22.a	Have EMS responders been trained how to use interagency communication protocols?	22	10	3	6
1.22.b	Has dispatch been trained on how to communicate with police dispatch to coordinate police assistance on EMS runs?	27	6	2	6
Count		859	499	203	120
Percent		51.1%	29.7%	12.1%	7.1%
Checklist Number	Phase 2. Traveling to the Scene	M.F.	L.F.	E.D.	Missing
*Dispatch*					
2.1	Does your department have dispatch protocols for when to launch additional resources to support scene safety?	33	6	0	2
2.1.a	Are dispatchers trained on when to launch additional resources?	24	10	2	5
2.1.b	Does your department have a policy requiring dispatchers to keep the caller on the line until EMS arrives to ensure the sharing of information?	21	10	9	1
2.2	Has your department operationalized a ‘flag’ in your dispatch system to alert EMS responders to previously known violent locations or individuals?	21	17	1	2
2.2.a	Are dispatchers trained in communicating that a ‘flag’ exists for previously violent locations or individuals?	25	9	1	6
2.2.b	Are EMS responders trained in confirming with dispatch if the location is a previously violent location or individual?	23	10	2	6
*En Route*					
2.3	Does your department have a policy regarding the use of lights and sirens (e.g., responder discretion, tiered response)?	37	2	0	2
2.3.a	Have EMS responders been trained on scenarios which require different uses of lights and sirens?	27	6	1	7
*Police Assist*					
2.4	Does your department have a policy for calls that require police assistance (e.g., dispatch to notify that police are en route, EMS responders have been trained to check for police en route)?	30	3	0	8
2.4.a	Have dispatch, police, and EMS responders been trained on police assist?	30	5	0	6
2.5	Does EMS receive police dispatch data on neighborhoods and locations that have known risks for violence?	8	16	9	8
2.5.a	Are dispatchers trained to know how to use these data to inform EMS responders about risk?	13	12	10	6
2.6	Does your department have the capability to share radio frequency with police?	20	9	3	9
2.6.a	Have EMS responders been trained on how and when to share radio frequency?	24	7	4	6
2.6.b	Have EMS responders been trained to understand police department terminology?	20	10	4	7
2.7	Are there adequate resources to have joint police and EMS response?	12	10	13	6
Count		368	142	59	87
Percent		56.1%	21.6%	9.0%	13.3%
Checklist Number	Phase 3. Scene Arrival	M.F.	L.F.	E.D.	Missing
*Body Armor*					
3.1	Does your department have a policy regarding body armor for EMS responders (e.g., ballistic vests, helmets, stab protection)?	19	16	4	2
3.1.a	Are EMS responders trained on how and when to don body armor properly and what weapons the armor protects against?	13	19	6	3
3.1.b	At a minimum, is the department’s policy compliant with national standards, such as the NFPA?	13	18	7	3
*Dispatch*					
3.2	Does your department have a policy in place for EMS responders to communicate scene conditions upon arrival?	38	1	0	2
3.2.a	Have EMS responders been trained on how and when to communicate scene conditions?	33	3	0	5
3.2.b	Has dispatch been trained on how to respond depending upon the update from EMS responders?	28	7	2	4
Staging					
3.3	Does your department have policies for staging ambulances during events with a strong potential for violence (e.g., underlying medical condition, drug and alcohol influence, domestic violence, suicide attempts, behavioral/mental health emergencies, civil unrest, active shooters, terrorism, etc.)	35	3	0	3
3.3.a	Have EMS responders been trained on staging and exiting protocols?	31	7	0	3
*Interagency Incident Command*					
3.4	Does your department have protocols on communicating field updates to dispatch and vice versa?	33	4	0	4
3.4.a	Has dispatch been trained on receiving and responding to field updates while fielding other calls?	25	12	1	3
3.4.b	Has dispatch been trained to communicate with necessary agencies if an update necessitates more EMS responders or police?	23	14	1	3
3.4.c	Does your department have a feedback mechanism for communication failures and breakdowns?	25	9	3	4
*Scene Assessment and Safety*					
3.5	Does your department have protocols and tools for scene assessment?	31	8	0	2
3.5.a	Have EMS responders been trained on protocols for scene assessment?	27	9	0	5
Count		374	130	24	46
Percent		65.2%	22.6%	4.2%	8.0%
Checklist Number	Phase 4. Patient Care	M.F.	L.F.	E.D.	Missing
*De-escalation*					
4.1	Does your department have Standard Operating Procedures [SOPs] for specific call types as it pertains to becoming a potential threat to EMS responders (e.g., underlying medical conditions, drug and alcohol influence, domestic violence, suicide attempts, behavioral/mental health emergencies, civil unrest, active shooters, terrorism etc.)?	27	10	2	2
4.1.a	Have EMS responders been trained on these SOPs and how to care for patients in these specific call types?	19	16	2	4
4.1.b	Have EMS responders been trained on how to protect themselves in these situations?	12	20	5	4
4.2	Does your department have training on assessing patients and bystanders, and their environment and immediate vicinity for threats (i.e., physical, mental, or metabolic conditions, egress routes, physical barriers for bystanders, cover and concealment, potential weapons, etc.)?	29	11	0	1
4.3	Does your department have policies on de-escalation techniques for various patient conditions (i.e., physical, mental, or metabolic conditions)?	16	23	1	1
4.3.a	Have EMS responders been trained on these de-escalation techniques?	9	26	2	4
4.4	Does your department have policies on when to call for backup at the earliest recognition of a threat?	34	4	0	3
4.4.a	Does your department have a graduated response to various levels of threat recognition, both from patient and bystanders?	20	18	0	3
4.4.b	Have EMS responders been trained on when to call for backup?	31	7	0	3
*Restraints and Self-defense*					
4.5	Does your department have policies on when to use restraints (i.e., chemical restraints, physical restraints), and what interagency involvement is needed (e.g., Police, Medical Control, etc.)?	28	10	1	2
4.5.a	Are EMS responders trained on when and how to use restraints when not in contact or without approval from medical control?	19	17	1	4
4.6	Does your department have a policy on self-defense?	12	17	10	2
4.6.a	Are EMS responders trained on self-defense techniques (e.g., breakaways, disarming, evasive actions, and less lethal tactics such as taser and mace)?	1	25	11	4
4.6.b	Have EMS responders been trained on city and state laws related to self-defense and what is an appropriate response per department policy?	7	19	11	4
4.7	Does your department have a policy regarding leaving the scene - with or without the patient - when EMS responders’ safety is at risk?	27	11	1	2
4.7.a	Have EMS responders been trained on this policy?	22	14	1	4
*Weapons-related Safe Actions and Practices*					
4.8	Does your department have policies regarding safe practices while administering care (e.g., if weapons are found on patient or bystander, etc.)?	27	9	3	2
4.8.a	Does your department train EMS responders on these safe practices?	18	16	3	4
4.8.b	Is dispatch trained to inform EMS responders if weapons are on scene?	25	8	4	4
4.8.c	Have EMS responders been trained on safe practices while in the ambulance and administering patient care?	24	11	2	4
4.9	Does your department have a policy on weapon discovery and securement when in transit?	19	13	4	5
4.9.a	Have EMS responders been trained on this policy?	14	18	4	5
4.10	Does your department use a standardized coded language to convey danger on scene with all relevant agencies (i.e., EMS, Police, Fire, Hospital, etc.)?	17	19	3	2
4.10.a	Are EMS responders trained in the coded language to notify dispatch of any concerns (e.g., crowd forming) and to call dispatch for backup (e.g., police assist, extra fire truck)?	22	14	1	4
4.10.b	Is dispatch trained in this coded language to safely communicate with EMS responders in the field?	21	14	1	5
4.10.c	Does your department have a ‘panic button’ mechanism in place when coded language or verbal communication is not an option?	25	9	2	5
*Transport and Transfer to the Hospital*					
4.11	Does your department have a policy regarding receipt of dangerous or violent patients in emergency department?	20	19	1	0
4.11.a	Have EMS responders been trained on this policy?	15	21	1	4
4.12	Is there a system in place to let the hospital know that an EMS responder has been injured?	36	3	2	0
4.13	Is there notification during handoff at the hospital to alert staff of patient or bystander violence?	33	5	2	1
Count		629	427	81	93
Percent		51.1%	34.7%	6.6%	7.6%
Checklist Number	Phase 5. Assessing Readiness to Return to Service	M.F.	L.F.	E.D.	Missing
*Readiness to Return to Service*					
5.1	Does your department have a policy that gives EMS responders and supervisors the autonomy to decide what they need physically and emotionally after a call, prior to returning to service (e.g., return to quarters, peer support, Critical Incident Stress Management (CISM), Employee Assistance Program (EAP), time off before return to service, seek religious counsel, etc.)?	12	22	7	0
5.1.a*	Are you tracking what options are used?	NA	NA	NA	NA
5.1.b	Do all EMS responders (from top-down: chief, supervisors, field personnel) receive recurrent training on how to recognize acute, cumulative, and chronic stress exposures from on-duty sources and their personal lives in themselves and others?	11	24	5	1
5.1.c	Do all EMS responders (from top-down: chief, supervisors, field personnel) receive recurrent training on how to reflect on stress of the job and the importance of reflection as professional practice (e.g., post-incident emotional assessment)?	10	23	7	1
5.1.d	Are there certain calls or circumstances that result in a mandatory wellness check-in?	19	14	7	1
5.1.e	Do EMS personnel receive training and resources to build personal resiliency to deal with stressors outside of work?	10	26	4	1
5.2	Does your department have a policy that allows for recovery from work to reflect at the end of a call (e.g., post-incident emotional assessment), have breaks for food, time to use the bathroom, or rest during their shift?	18	13	10	0
5.2.a	Does your department have a policy regarding under what circumstances a unit can be forcibly/automatically returned to service, and who has the authority to override such an action (i.e., dispatcher, EMS responder, supervisor)?	14	20	6	1
5.2.b	Have dispatchers been trained on when they can and cannot call an ambulance back in service from a break?	17	18	5	1
5.2.c	Have dispatchers been trained on when EMS responders can override a return to service decision?	15	18	7	1
5.2.d	Have EMS responders been trained on how to communicate breaks to dispatch?	25	11	4	1
*Physical and Psychological Injury Assessment*					
5.3	Does your department have a policy that outlines how to support an EMS responder (physically and emotionally) who has experienced verbal or physical violence?	18	17	4	2
5.3.a	Do supervisors have training in stress recognition and management?	9	27	4	1
5.3.b	Have supervisors received training on how to identify and respond to EMS personnel expressing a need for breaks, or those suffering from stress exposure?	12	25	3	1
5.3.c	Does your department have a policy that allows supervisors to encourage responders to seek help?	21	12	7	1
5.3.d	Are the necessary support services available to EMS responders (e.g., counseling, Stress First Aid, Critical Incident Stress Management (CISM), Employee Assistance Programs (EAP), peer support programs, Crisis Response Teams (CRTs), Chaplains, etc.)?	16	20	4	1
5.4	Does your department have a non-punitive policy that specifies that coworkers should notify their field officer/supervisor when their partner is showing signs of stress exposure, or has experienced violence/injury?	21	11	7	2
5.4.a	If yes, are coworkers able to report concerns anonymously?	7	3	1	30
5.5	Does your department have a policy that specifies that EMS responders should notify their supervisor when they have experienced verbal or physical violence with or without injury?	33	7	1	0
*Staffing Policy*					
5.6	Does your department have policies to increase EMS responder staffing to cover overworked EMS responders as needed (e.g., having two additional EMS responders per shift to provide relief/coverage)?	4	16	21	0
5.6.a	Does your department have adequate staffing to support overworked or vacant positions?	5	16	20	0
5.6.b	Does your department have an agile overtime policy that can be implemented when someone needs to be taken out of service for emotional/physical recovery?	10	18	13	0
5.7	Does your department have stress pay/mental health days (i.e., day/days off) available for EMS responders?	5	16	19	1
5.7.a	Does your department differentiate work-related stress as an injury or a personal illness?	10	17	11	3
5.7.b	Can personnel use sick leave for mental health days?	16	14	8	3
5.7.c	Does your department clearly communicate if sick days can be utilized as mental health days?	18	11	8	4
5.8	Does your department have a policy/procedure to rotate EMS responders from busy stations to less busy stations for recovery time?	12	14	15	0
5.8.a	If yes, is it voluntary or mandatory?	11	8	7	15
5.9	Does your department routinely rotate responders between EMS and fire duties to provide relief from EMS overwork?	9	16	15	1
5.10	Are EMS responders trained on how, when, and who to ask for support and specialized resources when in need of recovery from work?	20	16	5	0
Count		408	473	235	73
Percent		34.3%	39.8%	19.8%	6.1%
Checklist Number	Phase 6. Post- Event	M.F.	L.F.	E.D.	Missing
*Reporting*					
6.1	Does your department train on the importance of and methods associated with reporting violent events?	23	16	1	1
6.1.a	Does your department perpetuate a safe culture for reporting so that members will not be disrespected or dismissed for reporting a violent event (i.e., will all reports be treated with seriousness and respect)?	22	14	5	0
6.1.b	Does your department encourage the reporting of all incidents of violence (verbal or physical) to reporting systems like EMERG, Occupational Safety and Health Administration (OSHA) 300, National Firefighter Near Miss Reporting System (NFFNMRS), National Fire Incident Reporting System (NFIRS), state requirements, etc.?	21	16	4	0
6.1.c	Does your department investigate ways to administratively simplify multiple reporting systems to encourage reporting of violent events?	5	30	6	0
6.1.d	Does your department encourage reporting violence that leads to physical injury and a clear process that leads the EMS responder to report to Workers’ Compensation?	34	5	2	0
6.1.e	Does your department have a way to disseminate immediate and brief information describing the violence experienced by your members?	23	15	3	0
6.1.f	Does your department have a policy that protects an EMS responder’s time - either by going out of service or using overtime - so that they can easily report any acts of violence or exposure they experienced on a call, before they return to service and go on their next call?	16	18	7	0
6.1.g	Does your department training include guidelines and best practices for documentation (including appropriate terminology) that can help to support the EMS responder, should the EMS responder narrative be used in court proceedings (i.e., inclusive of appropriate documentation for use of force, self-defense, and restraints, etc.)?	19	20	2	0
6.1.h	Does your department train EMS responders with a checklist that describes what should be included in a patient care report narrative regarding on scene violence targeting responders?	23	16	2	0
6.1.i	Does your department train EMS responders on how to communicate with police or investigators regarding a violent incident, when appropriate?	17	22	2	0
6.1.j	Does your department have a policy for collecting data for when dispatch does not advise crews of appropriate staging? Is there a mechanism for reviewing this policy?	13	20	8	0
6.2	Does your department have a policy that dictates that dispatch will flag previously known violent locations as reported by EMS responders, and this information will be conveyed on future calls without inadvertently identifying individuals?	19	19	3	0
6.2.a	Does your department have a policy that the violence dispatch flag is included in the Quality Assurance and Quality Improvement (QA/QI) process?	17	19	3	2
6.2.b	Does your department have policies to regularly update the list of violent locations?	16	17	6	2
*Organizational Support*					
6.3	Does your department have accessible and timely medical oversight to clear responders to return to work without docking pay or missing shifts?	23	11	7	0
6.4	Does your department have a return to work policy that addresses long-term clearance by mental health professionals?	8	23	10	0
6.5	Does your department issue guidance (SOP/SOG) for dispatchers and supervisors on how to interact with an injured EMS responder (e.g., acknowledging the violent encounter and its impact, not blaming the EMS responder, asking if they need treatment or psychological assistance, informing EMS responders of all reporting options such as Workers’ Compensation (if necessary), and assisting them with pressing charges (if desired), asking for their perspective on how this could have been prevented and what departmental resources are needed, contacting or visiting injured EMS responders at their home or medical facility by the department or IAFF local, disseminating information back to the department, providing support to injured responder)	17	16	6	2
6.5.a	Do your department’s supervisors receive recurrent training on this checklist?	7	21	3	10
6.5.b	Does your department have a policy that dictates immediate supervisor actions as they relate to filing reports of violent incidents?	18	10	3	10
6.6	Does your department utilize informal After Action Reviews (AAR) following violent events (e.g., What was your mission? What went well? What might we have done differently? What could have gone better? Who needs to know? How could this have been prevented and what resources from the department are needed?)	28	7	4	2
6.6.a	Are lessons learned from the informal AAR shared in a way that protects the responder’s privacy	18	8	5	10
6.6.b	Does your department have a protocol in place for an AAR of calls that required notifications, updates, or emergency communications?	21	9	4	7
6.6.c	Is information that is gained after an AAR shared with the rest of the department?	21	16	1	3
6.6.d	Does your department change policy/SOPs from items learned in the AAR process?	10	18	6	7
6.7	Does your department measure organizational outcomes that are important to EMS responders (e.g., burnout, job satisfaction, engagement, intention to leave the profession, turnover)?	6	22	13	0
6.8	Does your department offer recurrent training to field supervisors and leadership on the importance of safety culture, safety outcomes, and organizational outcomes?	14	22	5	0
*Immediate Mental Health Support*					
6.9	Does your department have one or more post-incident support programs instituted for EMS responders who need them? (e.g., Stress First Aid, Critical Incident Stress Management (CISM), Employee Assistance Programs (EAP), peer support programs, Crisis Response Teams (CRTs), Chaplains, etc.)	22	14	5	0
6.9.a	Has your department identified a best practice for implementation of confidential mental health support (e.g., before returning to service, after returning to quarters, informally - when convenient and asked for by EMS responders)?	17	17	5	2
6.9.b	Has your department considered external resources to provide the appropriate level of support for post-incident needs (e.g., peer support group for high risk occupations, EMS responder-trained psychologists, etc.)?	17	16	6	2
6.9.c	Has your department considered using an outside agency to handle EAP (should not be in the same building as department administration)?	18	13	8	2
6.9.d	Has your department trained members on how to access these resources and/or best practices for implementation, should they need them?	26	9	4	2
*Long-Term Physical and Mental Health Support*					
6.10	Does your department have a policy that an Employee Assistance Program (EAP) representative, mental health counselor, city insurance case manager, etc., can perform mental health checks on injured EMS responders?	19	18	4	0
6.11	Are diverse modalities offered within the department for mental health support programs (e.g., Psychological First Aid, Critical Incident Stress Management (CISM), Complementary Alternative Medicine modalities (CAM), HeartMath, Mindfulness-based Stress Reduction programs (MSBR), etc.)?	13	21	7	0
6.12	Have EMS personnel, regardless of rank, been trained on recognizing signs of cumulative stress, paying particular attention to the long-term impact of this work?	16	22	3	0
6.13	Do those contracted to provide mental health services have demonstrated experience working with EMS responders?	10	23	8	0
6.13.a	Are these mental health services accepted by EMS responders?	4	13	2	22
6.14	Does your department provide recurrent training on adaptive skills, such as coping and resiliency?	11	24	6	0
6.15	Does your department’s training curriculum recognize and train on stress as a chronic occupational exposure, including the relationship between the EMS responder workload and its cumulative stress impact?	12	20	8	1
6.15.a	Are EMS personnel, regardless of rank, trained on stress as a chronic occupational exposure (i.e., trained on the physiological effects of stress, recognizing cumulative stress exposure in one’s self and others)?	14	15	8	4
*Support for Court*					
6.16	Does your department train EMS responders to know that your state has criminal statutes, should they be assaulted?	22	14	5	0
6.17	Does your department provide support and information about available resources for court to the assaulted EMS responder as they maneuver the court/legal system?	18	14	9	0
6.17.a	Does a member of your department, IAFF Local, or other advocate attend court with the assaulted responder?	17	19	3	2
6.17.b	Does your department have a policy that specifies that preparation for the judicial process and court appearances are compensable activities?	17	17	6	1
Count		732	719	218	94
Percent		41.5%	40.8%	12.4%	5.3%

* missing indicates non-response

# NA, not applicable because modification added after voting
