# Supplementary material for: Creation of a Systems-Level Checklist to Address Stress and Violence in Fire-Based Emergency Medical Services Responders
Source: Occup Health Sci. Author manuscript; Available in PMC 2021 Nov 17. (PMC8596461)
Supplement: ESM1 [file NIHMS1749898-supplement-ESM1.pdf]

Stress and Violence in fire-based EMS Responders (SAVER)
Systems Checklist Consensus Conference (SC^3)

ThinkLet 1: Idea Generation

Purpose:

This ThinkLet is for the initial idea generation session for the checklist. The large group will be divided into six groups of seven to eight individuals, and each of these groups will be in a breakout room. Each group will have a different phase of the checklist, and the individuals will be asked to review and generate ideas for the specific phase.

Room Set-up:

- Each breakout room will need the following
  - Markers and pens
  - Large notecards
  - Copies of checklist for that phase
  - Large flip pad

Outline for ThinkLet:

- Facilitator will tell participants each session will be recorded. Fellows will turn on recorder (placed in the center of the table), and remind facilitator to use their phone as a backup recording device. Backup recordings will be deleted once we confirm the recording for each breakout session is complete.
- Ensure recorder is on and functioning
- Introduce yourself and have the participants introduce themselves
- Reiterate the opening remarks and establish ground rules for the session (we will have the rules posted in the front of the room – use the flip chart for this)
  - The focus of this session is on the safety of the first responders
  - Everyone here has valuable experience to add
  - Respect the other individuals in the room
  - Be as honest as you feel comfortable as this will help us determine the best course of action in developing a comprehensive checklist
- ***Introduce the Phase that will be the focus of this ThinkLet (10 mins)***
  - Reiterate that over the course of the day they will be working with two other phases, but to only focus on this particular phase
  - Define the phase – be sure that it is showcased on the top of all materials and written on the boards
  - Present the participants with the current check-list items and have them review

        - "These checklist items were developed by a team of researchers that have reviewed practices from first responders across the nation. These represent the current best-practices that were available to our team"

- ***Idea Generation (25 minutes in small groups)***
    - Split the breakout group into two groups of 3-4 people
    - "Now that you have reviewed the current checklist, we would like your feedback regarding the list. We are going to split into two groups (3-4 people ea.). Try to work with individuals you have not met before (if at all possible). Consider the following questions:
        - What is missing from the list? Please write down each idea or item on an individual note card
        - What items need to be revised? Please write down the revised idea on an individual note card
        - Are there any items that should not be in this phase that are appearing on the checklist? Please cross them out on the item sheet. **Indicate if you are throwing the item out, or you feel that it does not belong in this phase**
    - "We will give you a five minute warning, and in the last five minutes, we would like your group to decide the top seven most critically missing items or revised items that you see. Please number them 1 (most critical) through 7 (least critical) on the notecard"
    - **Note for facilitators: if the conversation seems to be stagnating – prompt the groups to think of the different levels (e.g., policy, training, etc.)**
- ***Idea Discussion (20 minutes in breakout group)***
    - Bring the small groups back together
    - "Now that you have had a chance to work with your groups and rank the top seven items that need to be revised or added, please give those to the facilitator"
        - We should have roughly 15 items
    - Facilitator will read out the 15 items and place them in a central table.
    - "Now that you have heard the 15 items and they are placed here on the central table, what ideas do you feel are left out from this list that came up in your group? Where are the redundancies in this list that can be collapsed? Do you have a revised statement that the group can generate to refine the checklist item?"
- ***Debrief and Conclusion (5 minutes)***
    - Thank the participants and summarize what was discussed in the current session.
    - Provide instructions for where to go and what is to come – break for one hour, with ThinkLet 2 beginning at 1pm

Stress and Violence in fire-based EMS Responders (SAVER)
Systems Checklist Consensus Conference (SC^3)

ThinkLet 2: Idea Generation

Purpose:

This ThinkLet is the second idea generation session for the checklist. Each group of seven to eight individuals will be moved to a different breakout room that will be for a different phase of the checklist.

Room Set-up:

- Each breakout room will need the following
  - Markers and pens
  - Large notecards
  - Copies of checklist for that phase
  - Large flip pad

Outline for ThinkLet:

- Facilitator will tell participants each session will be recorded. Fellows will turn on recorder (placed in the center of the table), and remind facilitator to use their phone as a backup recording device. Backup recordings will be deleted once we confirm the recording for each breakout session is complete.
- Ensure recorder is on and functioning
- Introduce yourself and have the participants introduce themselves
- ***Introduce the Phase that will be the focus of this ThinkLet (10 mins)***
  - Reiterate that over the course of the day they will be working with two other phases, but to only focus on this particular phase
  - Define the phase – be sure that it is showcased on the top of all materials and written on the boards
  - Present the participants with the current check-list items and have them review
    - "These checklist items were developed by a team of researchers that have reviewed practices from first responders across the nation. These represent the current best-practices that were available to our team"
- ***Idea Generation (25 minutes in small groups)***
  - Split the breakout group into two groups of 3-4 people
  - "Now that you have reviewed the current checklist, we would like your feedback regarding the list. We are going to split into two groups of three to four. Try to work with individuals you have not met before (if at all possible). Consider the following questions:

        - What is missing from the checklist list and the items generated by your colleagues? Please write down each idea or item on an individual note card
        - What items need to be revised? Please write down the revised idea on an individual note card
        - Are there any items that should not be in this phase that are appearing on the checklist? Please cross them out on the item sheet. **Indicate if you are throwing the item out, or you feel that it does not belong in this phase**
    - "We will give you a five minute warning, and in the last five minutes, we would like your group to decide the top seven most critically missing items or revised items that you see. Please number them 1 (most critical) through 7 (least critical) on the notecard"
    - **Note for facilitators: if the conversation seems to be stagnating – prompt the groups to think of the different levels (e.g., policy, training, etc.)**
- ***Idea Discussion (20 minutes in breakout group)***
    - Bring the small groups back together
    - "Now that you have had a chance to work with your groups and rank the top seven items that need to be revised or added, please give those to the facilitator"
        - We should have roughly 15 items
    - Facilitator will read out the 15 items and place them in a central table.
    - "Now that you have heard the 15 items and they are placed here on the central table, what ideas do you feel are left out from this list that came up in your group? Where are the redundancies in this list that can be collapsed? Do you have a revised statement that the group can generate to refine the checklist item?"
- ***Debrief and Conclusion (5 minutes)***
    - Thank the participants and summarize what was discussed in the current session.
    - Provide instructions for where to go and what is to come.

Stress and Violence in fire-based EMS Responders (SAVER)
Systems Checklist Consensus Conference (SC[3])

ThinkLet 3: Idea Generation

Purpose:

This ThinkLet is the third idea generation session for the checklist. Each group of seven to eight individuals will be moved to a different breakout room that will be for a different phase of the checklist.

Room Set-up:

- Each breakout room will need the following
  - Markers and pens
  - Large notecards
  - Copies of checklist for that phase
  - Large flip pad

Outline for ThinkLet:

- Facilitator will tell participants each session will be recorded. Fellows will turn on recorder (placed in the center of the table), and remind facilitator to use their phone as a backup recording device. Backup recordings will be deleted once we confirm the recording for each breakout session is complete.
- Ensure recorder is on and functioning
- Introduce yourself and have the participants introduce themselves
- ***Introduce the Phase that will be the focus of this ThinkLet (10 mins)***
  - Reiterate that over the course of the day they will be working with two other phases, but to only focus on this particular phase
  - Define the phase – be sure that it is showcased on the top of all materials and written on the boards
  - Present the participants with the current check-list items and have them review
    - "These checklist items were developed by a team of researchers that have reviewed practices from first responders across the nation. These represent the current best-practices that were available to our team"
- ***Idea Generation (25 minutes in small groups)***
  - Split the breakout group into two groups of 3-4 people
  - "Now that you have reviewed the current checklist, we would like your feedback regarding the list. We are going to split into two groups of three to four. Try to work with individuals you have not met before (if at all possible). Consider the following questions:

        - What is missing from the checklist list and the items generated by your colleagues? Please write down each idea or item on an individual note card
        - What items need to be revised? Please write down the revised idea on an individual note card
        - Are there any items that should not be in this phase that are appearing on the checklist? Please cross them out on the item sheet. **Indicate if you are throwing the item out, or you feel that it does not belong in this phase**
    - "We will give you a five minute warning, and in the last five minutes, we would like your group to decide the top seven most critically missing items or revised items that you see. Please number them 1 (most critical) through 7 (least critical) on the notecard"
    - **Note for facilitators: if the conversation seems to be stagnating – prompt the groups to think of the different levels (e.g., policy, training, etc.)**
- ***Idea Discussion (20 minutes in breakout group)***
    - Bring the small groups back together
    - "Now that you have had a chance to work with your groups and rank the top seven items that need to be revised or added, please give those to the facilitator"
        - We should have roughly 15 items
    - Facilitator will read out the 15 items and place them in a central table.
    - "Now that you have heard the 15 items and they are placed here on the central table, what ideas do you feel are left out from this list that came up in your group? Where are the redundancies in this list that can be collapsed? Do you have a revised statement that the group can generate to refine the checklist item?"
- ***Debrief and Conclusion (5 minutes)***
    - Thank the participants and summarize what was discussed in the current session.
    - Provide instructions for where to go and what is to come.

Stress and Violence in fire-based EMS Responders (SAVER)
Systems Checklist Consensus Conference (SC^3)

ThinkLet 4: Convergence

Purpose:

This ThinkLet is held with the entire participant group, and is intended to be the first round of convergence. The large group will be separated into new groups of seven to eight individuals, but we will remain in the same room. The smaller groups will rotate between poster-boards, which will hold the generated checklist items from the day before.

Room Set-up:

- The central room should have:
    - Typed set of items and ideas from previous day on Poster-boards
    - Big Sticky Notes
    - Markers
    - Big flip pad
    - Blue-tac

Outline for ThinkLet:

- Facilitator will tell participants each session will be recorded. Fellows will turn on recorder (facilitator to hold near poster-boards and participants) and remind facilitator to use their phone as a backup recording device. Backup recordings will be deleted once we confirm the recording for each breakout session is complete.
- Ensure recorder is on and functioning
- Provide a recap of the previous day
- Reiterate the opening remarks and briefly touch on ground rules again
    - Ground rules should be posted in the room in a couple of locations

- ***Introduce the activity (5 minutes)***
    - "You have all gone through three rounds of producing and cleaning ideas for three separate phases of the checklist. We are now going to come to consensus on which items are going to be included in the specific phases. You will see that six-large poster-boards are spread around the room. Each poster-board contains one phase of the checklist, and all the items that were generated by the research team as well as your work yesterday. We will be working in 10-minute increments. You have been assigned to a new group different from the one you were in yesterday. In those ten minutes, we will be identifying which items should be included, where there are redundancies, and which items should be either removed or moved to another phase. This is intended to be quick and you will be working through all six phases over the next 90 minutes"

- ***Convergence activity (6 x 15 minute sprints- 90 mins)***
    - Facilitators will need to ensure that there is smooth flow and timing from phase to phase
    - Groups will move between poster-boards, and each poster will have a phase on it
    - Give participants 3 minutes warning
    - Provide any assistance writing wise (if a new idea comes up)

- ***Debrief (5 minutes)***
    - Thank the participants.
    - Dismiss the group for a networking break.

Stress and Violence in fire-based EMS Responders (SAVER)
Systems Checklist Consensus Conference (SC^3)

ThinkLet 5: Feasibility Assessment

Purpose:

This ThinkLet is held with the entire participant group. The individuals have now come to reasonable convergence across the six phases, and this ThinkLet will provide us with the different levels of feasibility on checklist items.

Room Set-up:

- The central room should have:
    - Red, Yellow, and Green stickers
    - Post the definitions of red, yellow, & green around room after voting is explained

Outline for ThinkLet:

- Recap what the individuals have completed up to this point
- ***Introduce final ThinkLet (5 minutes)***
    - "This final ThinkLet is intended to identify different levels of feasibility for checklist items. In short, we want to know which checklist items are considered easily implemented to address the safety of EMS responders, and which may be less feasible (although still important)"
- ***Red, Yellow, Green Voting (6 x 10 minute sprints- 60mins)***
    - "In this activity we will be voting on the checklist items using three colors – red, yellow, and green. You each will cast a vote on every checklist item in each phase. You will be voting individually, but we encourage you as you move around the room to discuss any issues that arise. Here is how you can assign the three colors:
        - ***Green – most feasible checklist item and easily implemented.*** These items appear to be the easiest items on the checklist for departments to implement in a reasonably short period of time (i.e. within 3- 6 months).
        - ***Yellow – less feasible checklist item, but still a reasonably accomplishable item***. These items are more difficult than green items in terms of feasibility and implementation, but still worth pursuing, particularly as the green items are accomplished (i.e., within 1 – 2 years).
        - ***Red – extremely difficult to implement or infeasible checklist item***. These items are believed to be nearly impossible to implement due to a number of factors (e.g. local, state, or federal regulations). However, should both green and yellow items be accomplished, further efforts may be made to eventually accomplish these more difficult items (i.e., 2 years - Never).
- ***Debrief (15 minutes)***
    - Thank individuals and recap the entire process to offer a conclusion for the two days
