# Supplementary material for: Creation of a Systems-Level Checklist to Address Stress and Violence in Fire-Based Emergency Medical Services Responders
Source: Occup Health Sci. Author manuscript; Available in PMC 2021 Nov 17. (PMC8596461)
Supplement: ESM2 [file NIHMS1749898-supplement-ESM2.pdf]

Feasibility Assessment of Full Checklist, Original Checklist Items, and Newly Generated Checklist Items

Phase 1. Pre-Event

Calculations of Full Checklist			Calculations of Original Checklist Items		%feas	Calculations of Newly Generated Checklist Items		%feas
%mf	859	51.1	%mf	521	**57.8**	%mf	338	**43.4**
%lf	499	29.7	%lf	254	**28.2**	%lf	245	**31.5**
%ed	203	12.1	%ed	69	**7.6**	%ed	134	**17.2**
%missing	120	7.1	%missing	58	**6.4**	%missing	62	**8.0**
Totals	1681	100.0	Totals	902	**100**	Totals	779	**100**

Phase 2. Traveling to the Scene

Calculations of Full Checklist			Calculations of Original Checklist Items			Calculations of Newly Generated Checklist Items		%feas
%mf	368	56.1	%mf	327	**57.0**	%mf	41	**50.0**
%lf	142	21.6	%lf	122	**21.3**	%lf	20	**24.4**
%ed	59	9.0	%ed	46	**8.0**	%ed	13	**15.9**
%missing	87	13.3	%missing	79	**13.8**	%missing	8	**9.8**
Totals	656	100.0	Totals	574	**100.0**	Totals	82	**100.0**

Phase 3. Scene Arrival

Calculations of Full Checklist			Calculations of Original Checklist Items			Calculations of Newly Generated Checklist Items		%feas
%mf	374	65.2	%mf	336	**68.3**	%mf	38	**46.3**
%lf	130	22.6	%lf	103	**20.9**	%lf	27	**32.9**
%ed	24	4.2	%ed	14	**2.8**	%ed	10	**12.2**
%missing	46	8.0	%missing	39	**7.9**	%missing	7	**8.5**
Totals	574	100.0	Totals	492	**100.0**	Totals	82	**100.0**

Phase 4. Patient Care

Calculations of Full Checklist			Calculations of Original Checklist Items			Calculations of Newly Generated Checklist Items		%feas
%mf	629	51.1	%mf	516	**52.4**	%mf	113	**45.9**
%lf	427	34.7	%lf	329	**33.4**	%lf	98	**39.8**
%ed	81	6.6	%ed	69	**7.0**	%ed	12	**4.9**
%missing	93	7.6	%missing	70	**7.1**	%missing	23	**9.3**
Totals	1230	100.0	Totals	984	**100.0**	Totals	246	**100.0**

Phase 5. Assessing Readiness to Return to Service

Calculations of Full Checklist			Calculations of Original Checklist Items			Calculations of Newly Generated Checklist Items		%feas
%mf	408	34.3	%mf	215	**35.0**	%mf	193	**33.6**
%lf	473	39.8	%lf	249	**40.5**	%lf	224	**39.0**
%ed	235	19.8	%ed	143	**23.3**	%ed	92	**16.0**
%missing	73	6.1	%missing	8	**1.3**	%missing	65	**11.3**
Totals	1189	100.0	Totals	615	**100.0**	Totals	574	**100.0**

Phase 6. Post-Event

Calculations of Full Checklist			Calculations of Original Checklist Items			Calculations of Newly Generated Checklist Items		%feas
%mf	732	41.5	%mf	610	**42.5**	%mf	122	**37.2**
%lf	719	40.8	%lf	601	**41.9**	%lf	118	**36.0**
%ed	218	12.4	%ed	168	**11.7**	%ed	50	**15.2**
%missing	94	5.3	%missing	56	**3.9**	%missing	38	**11.6**
Totals	1763	100.0	Totals	1435	**100.0**	Totals	328	**100.0**

Total Calculations of Original Checklist Items			Total Calculations of Newly Generated Checklist Items		%feas
%mf	2525	**50.5**	%mf	845	**40.4**
%lf	1658	**33.1**	%lf	732	**35.0**
%ed	509	**10.2**	%ed	311	**14.9**
%missing	310	**6.2**	%missing	203	**9.7**
Totals	5002	**100.0**	Totals	2091	**100.0**
